# Aerosol-Assisted Plasma Deposition of Quercetin-Containing Coatings for Active Packaging Applications

**DOI:** 10.1021/acsomega.6c03125

**Published:** 2026-07-08

**Authors:** Angelica Maria Lanza, Antonella Milella, Nicoletta De Vietro, Giovanna Mancini, Fabio Palumbo, Donatella Nardiello, Francesco Fracassi, Carlo Zambonin, Pietro Favia

**Affiliations:** † Department of Chemistry, 9295 University of Bari “Aldo Moro”, via Orabona 4, Bari 70125, Italy; ‡ Institute of Nanotechnology, CNR, c/o Department of Chemistry, University of Bari “Aldo Moro”, via Orabona 4, Bari 70125, Italy; § Department of Biosciences, Biotechnology and Environment, 9295 University of Bari “Aldo Moro”, via Orabona 4, Bari 70125, Italy; ∥ Department of Agriculture, Food, Natural Resources and Engineering (DAFNE), 18972 University of Foggia, via Napoli 25, Foggia 71122, Italy

## Abstract

Quercetin (*QC*)-containing coatings were prepared by aerosol-assisted atmospheric-pressure plasma deposition employing two distinct strategies. In the first approach, *QC* was deposited by solution drop casting, followed by the deposition of a plasma-deposited barrier layer; in the second, the active compound was directly incorporated into the coating via plasma deposition from a quercetin-based aerosol feed. The two deposition strategies were comparatively evaluated in terms of chemical composition and surface morphology. The effects of *QC* concentration in the drop-cast or in the aerosol precursor solutions and of the thickness of the coating were investigated on the film composition and antioxidant performances. Both strategies led to the incorporation of quercetin in the final coating. HPLC and DPPH assays revealed that plasma exposure degrades *QC*, but the bilayer approach preserves up to 30% of its antioxidant activity. These findings pave the way for the scalable, solvent-free fabrication of bioactive coatings.

## Graphical abstract

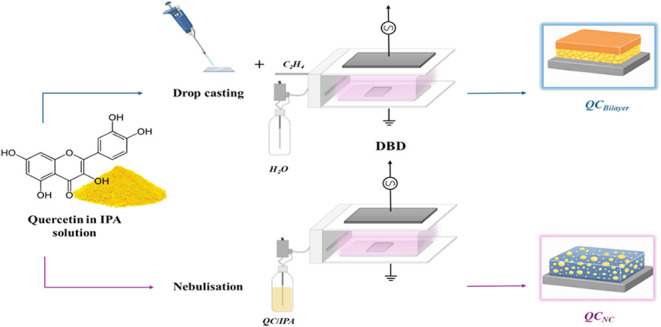

## 1 Introduction

Packaging plays a crucial role in protecting food products from external environmental factors during the supply chain. In response to evolving market trends, there is an increasing demand from consumers, retailers, and producers for fresh, high-quality, minimally processed, and long-shelf-life food products. To address these demands, innovative packaging strategies have been developed, including active packaging materials.^1^ Such systems are designed to interact with food products, enhancing their quality and extending the shelf life through the incorporation of active components that either release beneficial compounds into or scavenge harmful compounds from the packaging environment. These active agents may include antimicrobials, antioxidants, ethylene and oxygen scavengers, as well as carbon dioxide emitters.^2,3^

Besides microbial growth, lipid oxidation represents a major cause of food spoilage. This process leads to the formation of toxic aldehydes, the degradation of unsaturated fatty acids, and the development of off-flavors associated with rancidity. Antioxidants are commonly added to food products to inhibit oxidative degradation. However, the increasing preference of consumers for healthier additive-free foods, together with the limitations of vacuum and modified-atmosphere packaging technologies, has driven growing interest in antioxidant-based active packaging solutions.^4^

Synthetic antioxidants such as butylated hydroxytoluene and butylated hydroxyanisole have been widely utilized since long time in the food industry due to their stability, availability, and low cost. Nevertheless, concerns regarding their potential health risks have prompted a shift in the direction of natural antioxidants, particularly toward phenolic compounds. These secondary plant metabolites are abundant in plant-based foods and include tocopherols, flavonoids, and phenolic acids.^5^ Quercetin (3,5,7,3′,4′-pentahydroxyflavone), whose structure is shown in Figure 1, in particular, from now on *QC*, among other flavonoids, exhibits particularly high antioxidant activity.^6^ *QC* is largely found in fruits, vegetables, and leafy greens, especially onions, apples, and capers, as well as in various plant tissues such as bark, stems, leaves, and roots.^7^ In addition to its antioxidant properties, *QC* has been reported to exhibit antimicrobial,^8^ antiviral,^9^ anticancer,^10^ neuroprotective,^11^ anti-inflammatory,^12^ cardiovascular,^13^ and antiobesity activities.^14^ For all these properties,^15,16^ Quercetin is classified as a Generally Recognized As Safe (GRAS) substance and is widely used in dietary supplements.^15^

**Figure 1:**
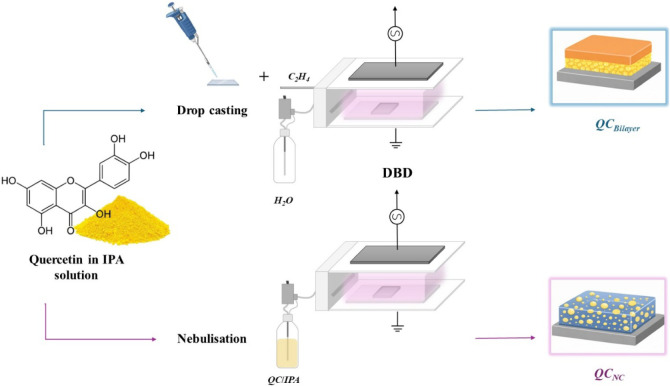
Quercetin structure and schematic representation of *QC*_bilayer_ and *QC*_NC_ deposition approaches.

In recent years, extensive research has focused on the development of *QC*-based active packaging systems.^16^ Functional films exhibiting antioxidant, antimicrobial, and UV-protective properties have been fabricated by incorporating *QC* into natural polymer matrices.^17^ Studies have demonstrated that the incorporation of *QC* into nanoparticle- and nanofiller-based films effectively preserved grapes^18^ and meat products.^19^ In addition, *QC*–chitosan composite systems have been extensively investigated for their synergistic antioxidant and antibacterial effects.^19–22^

Non-equilibrium Plasma Technologies, where plasmas are defined as ionized gases including excited molecules, atoms, ions, and free electrons, offer several processes for the modification of material surfaces for many applications and products in Microelectronics, Textiles, Biomaterials, Energy, and Packaging, to mention just a few areas.^23,24^ A promising strategy for the fabrication of active packaging materials involves the deposition of surface coatings containing the active molecule of interest (e.g., *QC*). Cold plasma thin-film deposition has emerged as an effective approach for this purpose, as it enables the treatment of both natural and synthetic polymers under ambient conditions, thereby preserving the integrity of thermosensitive substrates.^25,26^ In particular, Aerosol-Assisted Plasma Deposition (AAPD) at atmospheric pressure allows the incorporation of thermodegradable or low-volatility compounds, including bioactive molecules^27,28^ into coatings from liquid solutions or dispersions. This technique enables the deposition of inorganic–organic nanocomposites^29,30^ as well as nanobiocomposite coatings,^28,31^ while offering advantages such as high deposition rates and reduced processing costs due to the limited need for vacuum systems.^32^

Previous studies by our group have successfully demonstrated the feasibility^32^ of this approach for the incorporation of antibiotic molecules, such as lysozyme, vancomycin, and gentamicin, embedded in plasma-deposited coatings. It has been shown that such matrices enabled controlled release and provided protection of the drugs from the action of the plasma, while their biological antibiotic activity is retained.^33,34^

Given the growing interest in *QC*-based systems for active food packaging systems, this research was organized to investigate the feasibility of cold plasma processes to fabricate antioxidant *QC*-containing films. Specifically, bilayer and composite films were deposited using AAPD processes in a dielectric barrier discharge (DBD) plasma reactor. The chemical and morphological characteristics of the films were analyzed, and their antioxidant properties were systematically evaluated.

## 2 Materials and Methods

### 2.1 Materials

Helium 99.999% and ethylene 99.95% (Air Liquide, Milan, Italy) were used to feed the DBD discharges for the deposition processes. A solution of *QC* dihydrate (≥95%, Farmalabor, BAT, Italy) in 2-propanol (IPA) (≥99.8% ITW Reagents, Milan, Italy) was used for drop casting or in the aerosol feed of the film deposition process. Shards (1.5 × 1.5 cm^2^) of 625 μm thick double-faced polished crystalline silicon (100) wafers (MicroChemicals GmbH, Ulm, Germany) and squares (4 cm^2^) of 100 μm thick PET (Goodfellow) were used as substrates. To evaluate the antioxidant activity, 2,2-diphenyl-1-picrylhydrazyl (DPPH) and analytical grade methanol (≥99.9% , Sigma-Aldrich, Milan, Italy) were utilized.

### 2.2 DBD Plasma Reactor

The home-built DBD reactor used to run the AAPD deposition processes was extensively described in previous works.^33,35^ In brief, it consists of a Plexiglas housing containing two 5 × 8 cm^2^ silver electrodes covered with a 0.63 mm alumina layer. The electrodes are arranged in a parallel configuration with a gap of 3 mm among them. The upper electrode is connected to the power supply, while the lower electrode is grounded and serves as a sample holder. The discharges were triggered between the electrodes in continuous mode using a broadband AC power amplifier (AL-1000-HF-A, AMP-LINE Corp.) driven by a function generator (TG-1000, TTi Inc.) and a high-voltage transformer (AL-T1000, AMP-LINE Corp.). The current supplied to the system and the voltage were measured with a resistive (home-built) probe and a high-voltage probe (P6015A, Tektronix Inc.), respectively. To monitor the electrical characteristics of the system, both probes were connected to an oscilloscope (TDS 2014C, Tektronix). The aerosol of water and *QC* solutions was generated with a constant-output pneumatic nebulizer (model 3067, TSI) fed with He; two auxiliary lines fed the reactor with other He and ethylene. The gas flow rates were measured with mass flow meters (MSK Inc.). Prior to entering the reactor, the gas and aerosol stream passed through a mixing chamber to ensure homogeneous gas composition. The chamber was purged with 5 slm of He for 5 min before and after each deposition.

### 2.3 *QC*-Based Film Deposition Processes

Two coating approaches have been investigated: (i) a bilayer strategy, in which a *QC* layer is first deposited onto the substrate and subsequently coated with a plasma-deposited film acting as a barrier to slow down the release of *QC* (*QC*_bilayer_); (ii) a direct deposition approach of a nanocomposite coating embedding *QC* (*QC*_NC_). A schematic representation of the two processes is shown in Figure 1.

For the deposition of *QC*_bilayer_ films, 30 μL of *QC* solution was first drop-cast onto silicon substrates at concentrations of 0.50, 0.667, and 0.833 mg/mL in iso-propanol (IPA), corresponding to 15, 20, and 25 μg of *QC*, respectively. The solutions were prepared by diluting a 3.3 mg/mL stock solution of *QC* in IPA and stored in the dark at 4 °C. After the casting of QC, the samples were left at room temperature for 1 h before being plasma-coated with barrier films. The plasma deposition was carried out in a DBD fed with ethylene (30 sccm) and a water aerosol generated with 4 slm He, resulting in a mass flow rate of water aerosol of 100 mg/min. Barrier layers were deposited for 2, 6, and 10 min, resulting in film thicknesses as summarized in Table S1. For clarity, these coatings will hereafter be referred to as 200 nm, 600 nm, and 1 μm thick, corresponding to deposition times of 2, 6, and 10 min, respectively.

Control samples drop-casted *QC* with no barrier coating were also produced (*QC*_casted_). The same process was used to deposit a layer of 25 μg of drop-casted *QC*, a 600 nm barrier layer, and a bilayer comprising the two previous films onto a PET substrate.

The deposition of *QC*_NC_ films was achieved by feeding the plasma with an aerosol generated from a solution of *QC* in IPA. In this approach, the solvent itself was expected to provide reactive species upon the plasma fragmentation action for the formation of a matrix capable of embedding *QC* molecules. The aerosol feed solutions were prepared with concentrations in the range of 1.0–4.5 mg/mL; the deposition time was in the range of 15–30 min. The DBD was fed with 4 slm of He for the aerosol generator, with 2 slm of auxiliary He added. The mass flow rate of the aerosol feed under these conditions ranges from 300 to 620 mg/min, increasing as the *QC* concentration of the feed solution increases. Control films without *QC* were also deposited under the same conditions.

In both plasma deposition processes, samples were prepared in triplicate at a peak-to-peak voltage of 5 kV and at a frequency of 20 kHz. The average power was obtained by multiplying the energy per voltage cycle by the frequency of the applied field; the energy per cycle was calculated from the time integral of the current times the voltage in one cycle. Under the conditions explored, the power density was 1.2 W/cm^2^ regardless of the considered feed.

To better understand the effect of plasma exposure on *QC* and, in turn, to rationalize the results obtained for the deposition of the *QC*-containing coatings, films with 20 μg of the drop-casted *QC* were exposed to DBD ignited under the conditions described in Table 1. In these experiments, duration, peak-to-peak voltage, and frequency were kept constant at 10 min, 5 kV_pp_, and 20 kHz, respectively. Once prepared, the samples were stored in evacuated plastic boxes at 4 °C.

**Table 1:** Experimental Conditions to Test the Effect of Plasma on *QC*

Sample	He (slm)	Aerosol H_2_O (slm)	C_2_H_4_ (sccm)
He plasma	2	-	-
H_2_O/He plasma	2	4	-
C_2_H_4_/He plasma	2	-	30
H_2_O/C_2_H_4_/He plasma	2	4	30

## 3 Chemical and Morphological Characterization

### 3.1 Fourier Infrared Transmission Spectroscopy (FTIR) of Deposited Coatings

The FTIR characterization of the coatings was performed on polished silicon substrates (32 scans for analysis, at a resolution of 4 cm^–1^) with a Vertex 70 V spectrometer (Bruker). Prior to each analysis, the instrument was evacuated to pressures below 10 hPa. The FT-IR spectra were subsequently normalized with the thickness of the samples.

### 3.2 Film Thickness and Roughness Evaluation

The film thickness was determined from the step edge created by scratching the coatings deposited on the silicon substrates. The surface roughness was measured directly on the film surface. Both measurements were carried out using an AlphaStep D-120 profilometer (KLA-Tencor Instruments). The thickness and roughness of each coating were calculated as the averages of three independent measurements.

### 3.3 Film Wettability

To evaluate the wettability of the coatings, Water Contact Angle (WCA) measurements were performed with an OCA115EC digital goniometer (DataPhysics Instruments) using Milli-Q water 2 μL drops. Each WCA value was calculated as the average of three measurements.

### 3.4 Scanning Electron Microscopy (SEM)

Scanning electron microscopy measurements were performed with a ZEISS Sigma microscope (Carl Zeiss Co., Oberkochen, Germany), operating in the 0.5–20 kV range, with an in-lens secondary electron detector. Samples were mounted onto aluminum stubs with double-sided conductive carbon tape and grounded by means of silver paste.

### 3.5 Evaluation of *QC* Content: HPLC Analysis

The analytical quantification of *QC* in the deposited films was carried out by HPLC. For sample quantification, the films were dissolved in 2 mL of methanol (MeOH) via ultrasonication for 10 min. The solutions obtained were then diluted with the mobile phase before injection.

HPLC analysis was carried out with a high-performance instrument (HPLC; Series 30 HPLC system, Shimadzu, Milan, Italy) equipped with two Nexera LC-30AD pumps and a photodiode array UV–vis detector (SPD-20M20A, Shimadzu). Chromatographic separation was achieved on an Accucore C18 column (150 mm × 4.6 mm, 4 μm; Thermo Scientific, Milan, Italy), preceded by an Accucore C18 guard column (Thermo Scientific) to extend the lifetime of the column.

The mobile phase consisted of a mixture of water, methanol, and ethanol (56:40:6, v/v/v) containing 1.2% acetic acid. All solvents used were of HPLC grade (Sigma-Aldrich). Before the analysis, the mobile phase was filtered through a 0.45 μm nylon membrane (Lab Service Analytica, Bologna, Italy). Elution was conducted in isocratic mode at a flow rate of 0.8 mL/min.

The UV spectrum was recorded over the 220–500 nm range. *QC* detection and quantification were performed by monitoring the absorbance at 370 nm, corresponding to the compound’s characteristic absorption maximum of the compound.^36^ Data processing was carried out using LabService software, version 5.03.

Stock solutions of *QC* (1 mg/mL) were prepared in ethanol and stored at −20 °C in the dark. Working solutions (5 × 10^–4^–5 μg/mL) were obtained by appropriate dilution with the mobile phase and analyzed in triplicate using a UV-DAD detector to set up the calibration curve with the following equation:

y=12839x−199

where *x* represents the injected amount (ng) and *y* is the peak area. The method demonstrated excellent linearity over the concentration range investigated, with the correlation coefficient, R^2^, of 0.9985.

The limit of detection (LOD), calculated with a signal-to-noise ratio (S/N) of 3, and the limit of quantification (LOQ), with an S/N of 10, were found to be 0.1 ng and 0.3 ng, respectively. The precision of the method, expressed as relative standard deviation (RSD%), was evaluated by analyzing three standard solutions at known concentrations (0.005, 0.05, 0.5 μg/mL). Both intraday (*n* = 3) and interday (*n* = 3 on 5 consecutive days) precision were below 3.0% for all tested levels.

*QC:* Typical chromatograms obtained for the standard and for recovered *QC* are reported in the Supporting Information (Figure S1).^37^

### 3.6 UV–Vis Spectroscopy

UV–vis absorption spectroscopy was carried out to evaluate the spectrum of *QC* in the samples. As for HPLC, the films were dissolved in 2 mL of MeOH by ultrasonication for 10 min, and then, absorption spectra of the resulting solutions were collected with a Cary 60 UV–vis spectrophotometer (Agilent) in the range of 200–600 nm.

### 3.7 DPPH Assay for the Antioxidant Property

The antioxidant power of the coatings was assessed by means of a modified version of the DPPH assay reported by Santos et al.^37^ Films deposited on silicon shards were dissolved in 2 mL of methanol and sonicated for 10 min. Subsequently, 250 μL of the solution was added to 1.25 mL of a DPPH solution in methanol (0.01 mmol/L). The resulting mixture was kept at room temperature for 30 min in the dark; the absorption value was then recorded at 517 nm. All measurements were made in triplicate. The DPPH antioxidant activity, AOX_DPPH_ (%), was calculated with the following formula:

$$AOX_{\mathrm{DPPH}}\left(\%\right)=\frac{\left(A_{0}-A_{i}\right)}{A_{0}}\times 100$$

where *A*_0_ is the absorbance of the control (without *QC* addition) and *A*_i_ is the absorbance of the sample.

## 4 Results and Discussion

### 4.1 *QC*-Containing Coatings: Bilayer Approach

For this system, an organic coating containing polar groups, previously optimized as described in the work of Yang Y. et al., was used as a barrier.^38^ The mean deposition rate of such a barrier film was found to be 99 ± 4 nm/min (Table S1), with surface wettability being slightly hydrophilic, characterized by a WCA value of 68 ± 2° (Figure S2). As described in the previous section, coatings of different thicknesses were deposited by increasing the process time onto increasing amounts of drop-casted *QC*. For the same volume deposited, the thicknesses of the drop-casted *QC* layers are 60 ± 34 nm, 158 ± 73 nm, and 189 ± 37 nm for 15 μg, 20 μg, and 25 μg of *QC*, respectively.

The structural characteristics of the bilayer films were probed with FTIR spectroscopy, and the corresponding spectra are reported in Figure 2A together with those of the barrier coating and *QC*_casted_. The reported spectra correspond to an amount of *QC* of 25 μg. Changing the amount of *QC* resulted in no major changes, besides the intensity of the IR bands. The FTIR spectrum of the barrier layer exhibits absorption bands typical of O-containing hydrocarbon organic films, specifically in the regions 2958–2874 cm^–1^ and 1470–1380 cm^–1^, relative to alkyl groups. Additionally, oxygenated functional groups carbonyl (1705 cm^–1^) and hydroxyl (3440 and 1050 cm^–1^) were identified, accounting for the hydrophilic nature of the film. The band at 1050 cm^–1^ can also be attributed to ether moieties. In contrast, the FTIR spectrum of *QC* displays a broad absorption band attributed to the OH stretching at 3270 and 3400 cm^–1^, along with the highly intense absorption region in the range 1650–1010 cm^–1^, associated with carbonyl and hydroxyl groups, as well as with aromatic ring vibrations.^39^ As expected, the spectra of the bilayer samples contain all bands previously described, with the intensity of *QC*-related bands diminishing as the thickness of the deposited film increases.

**Figure 2:**
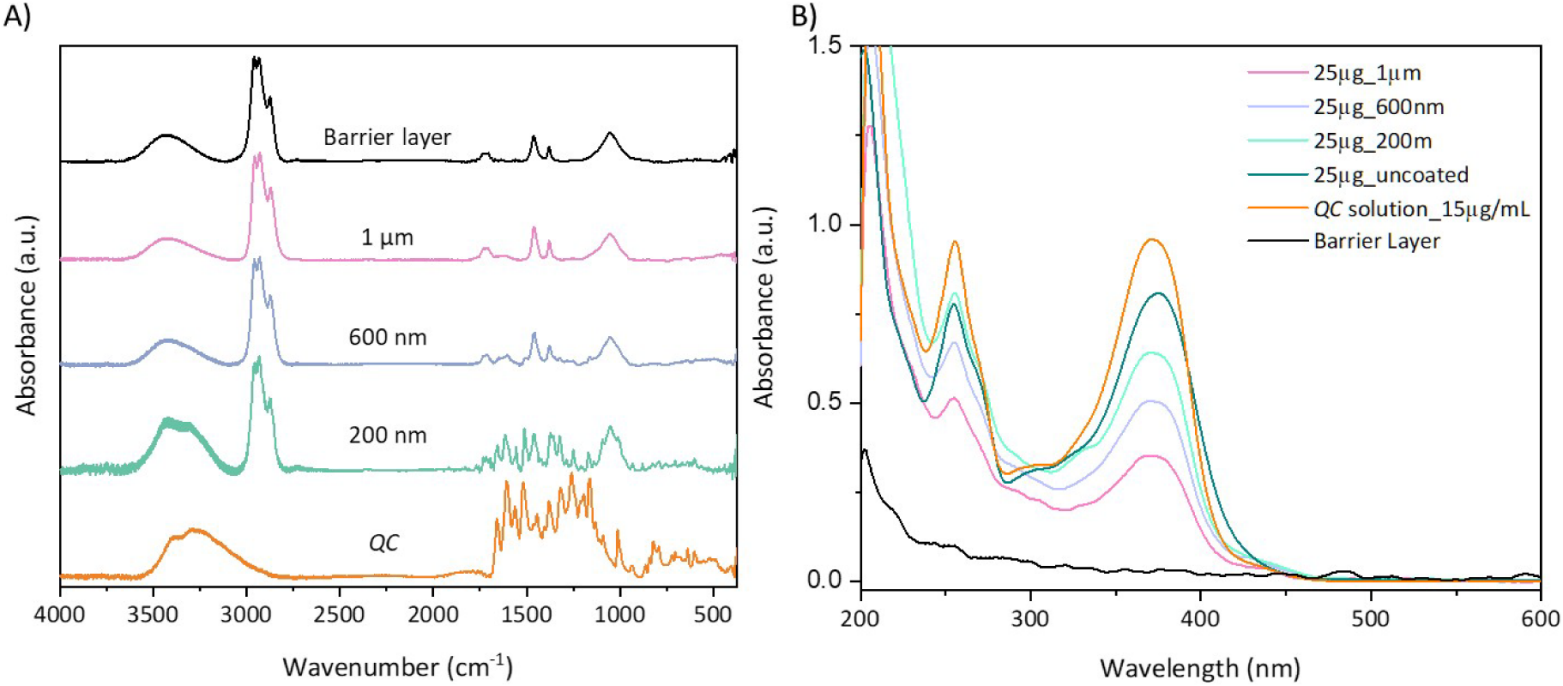
A) FTIR spectra of 25 μg of *QC*_casted_, *QC*_bilayer_ films with a barrier layer of different thickness and a 600 nm thick barrier layer. B) UV–vis spectra of 2 mL of MeOH after soaking *QC*_casted_ and *QC*_bilayer_ films prepared with 25 μg of quercetin, with a barrier layer of different thickness and a 600 nm thick barrier layer (the spectrum of a 15 μg/mL *QC* MeOH solution is reported for comparison).

Further characterization was achieved by dissolving films in 2 mL of MeOH and analyzing the obtained solutions using a UV–vis spectrophotometer. The resulting spectra are shown in Figure 2B. For comparison, the spectrum of a 15 μg/mL solution of pure *QC* in MeOH was acquired. All spectra show the characteristic absorption bands of *QC* at 372 and 255 nm^40^ except for the barrier layer, which exhibits only slight absorption below 250 nm. As is evident, the shape of the spectra remains almost unaltered irrespective of the presence or absence of the barrier film and its thickness. However, as the thickness of the film increases, a progressive decrease in the intensity of the characteristic peaks can be observed, despite the initial *QC* quantity being the same. Furthermore, the weak band present at 300 nm seems to disappear.

Samples were dipped and sonicated in methanol to extract quercetin (*QC* recovered amount) for its quantification at HPLC. Data have been reported in Table 2. As can be observed, the amount of *QC* is lower than the presumed amount deposited on the sample, even for the uncoated film (about 13 vs 25 μg). Two other main observations can be drawn: (i) the amount of unrecovered *QC* increases with the thickness of the barrier; (ii) the amount of recovered *QC* slightly increases with the amount of drop-casted quercetin. It appears, hence, that the thickness of the deposited film, and/or the processing time, influences the amount of *QC* recovered from the sample.

**Table 2:** Amount of *QC* Recovered from the Uncoated Drop Casted *QC* and Bilayers (on 1.5 × 1.5 cm^2^ Silicon Substrates) Evaluated by HPLC, Antioxidant Activity of the Different Bilayers Loaded with *QC,* and the Theoretical Antioxidant Activity Calculated Using the Amount of *QC* Determined by HPLC

Sample	*QC* Recovered Amount (μg)	Antioxidant Activity (%)	Estimated Antioxidant Activity (%)
25 μg _uncoated	12.6 ± 0.8	38 ± 2	13.1 ± 0.1
25 μg _200 nm	6.2 ± 0.8	30 ± 2	5.2 ± 0.3
25 μg _600 nm	3.4 ± 0.6	25 ± 2	1.7 ± 0.8
25 μg _1 μm	3.4 ± 0.6	18 ± 1	1.7 ± 0.8
20 μg _1 μm	2.0 ± 1	16 ± 1	0.3 ± 0.4
15 μg _1 μm	2.2 ± 0.8	13 ± 1	0.4 ± 0.1

This outcome points out a degradation of the *QC* molecule both as an effect of exposure to plasma (coated samples) and of the storage (uncoated samples). *QC*, indeed, is known to be relatively unstable due to the presence of a pyranoid ring and many OH groups in its structure. The stability of flavonoids is known to vary with the number of hydroxyl groups in the molecular structure, which, in this case, is three. *QC* degradation can be induced by various factors. High temperature and basic pH trigger the degradation of the molecule by oxidation, hydroxylation, and ring cleavage.^41^ *QC* is also photodegradable: when an alcoholic or alkaline solution of *QC* is exposed to UV radiation, a water or alcohol molecule is added to the double bond. In this case, the type of solvent makes a difference: the degradation of *QC* increases with the nucleophilic character of the solvent.^42^ Furthermore, the formation of polymerized products has been observed in alcoholic solvents, whereas in water, which is poorly solvating, and due to hydrogen interactions, oxidative degradation is more likely to occur.^43^ These considerations are not always taken into account when investigating new active packaging systems based on *QC*s. However, they can, in part, explain the lower amount of *QC* found in the samples studied in this work. Except during preparation, *QC* in *QC*_casted_ samples was dissolved in IPA and stored in the dark at low temperatures, while methanol was used for both quantification and UV–vis spectral recording. Under such conditions, one can expect the formation of polymerization products, in agreement with Pinelo et al.^43^ This should provide a plausible explanation for the lower amount of *QC* recovered from uncoated samples compared to the theoretical loading, highlighting the impact of solvent-induced degradation.

When looking at the barrier-coated samples, it should be considered that the drop-casted *QC* is exposed to plasma. During the discharge, the substrate is exposed to UV radiation and reactive species such as ions and radicals.^44^ Certainly, these interactions, in addition to those with solvents, can somewhat degrade quercetin molecules, resulting in the formation of various byproducts. Since the latter have a different absorption profile with respect to that of *QC*, a decrease in absorbance results. This is confirmed by the amount of *QC* found by HPLC analysis, which is always lower than those theoretically deposited. Furthermore, the above considerations lead to suppose that the observed *QC* diminution with the thickness of the barrier can be ascribed to the increasing exposure time to the plasma.

*QC* is a potent antioxidant, and it tends to react rapidly with DPPH via a sequential proton-loss electron transfer reaction.^45^ The results of the DPPH test are listed in Table 2. As expected, *QC*-free samples show no antioxidant activity, whereas the bilayers with *QC* do. As can be seen, the bilayers show antioxidant activity in the range of 13–30%. The antioxidant capacity exhibits an upward trend as the drop-casted amount of *QC* increases and the thickness of the barrier layer decreases. The latter follows the concentration trend of recovered *QC*, which, as explained previously, decreases as the plasma exposure time increases.

The measured amount of recovered *QC* determined by HPLC can be used to estimate the theoretical antioxidant activity of each sample using the calibration curve reported in Figure S3. These values are reported in Table 2 for comparison. Interestingly, it can be observed that the theoretical antioxidant activity is always lower than the measured one. In the case of *QC*_casted_, this effect is likely due to the mentioned formation of partially polymerized phenolic degradation products, which initially lead to a peak in antioxidant activity.^43^ Actually, the antioxidant efficacy is increased by polymerization because there is an increase in hydroxyls and extensive conjugation.^46^ For bilayers, however, the impact of plasma on *QC* must also be taken into account in addition to this effect, as it could lead to the formation and/or transformation of *QC* into byproducts with antioxidant activity.

### 4.2 *QC*-Containing Coatings: Direct Deposition of *QC* Composite Films

Since in this process *QC* is added continuously during the deposition, thicker films contain more *QC* for the same feed. To obtain a reliable comparison, it was decided to keep the thickness of the films at 500 nm, scaling the deposition time with the corresponding deposition rate. In Figure 3A, the FTIR spectra of *QC*_NC_ films deposited in one single step are reported. As can be seen, the *QC*-free matrix deposited with the isopropanol aerosol exhibits the same spectral bands as the barrier film deposited for the bilayer samples. Conversely, films incorporating *QC* demonstrate additional absorption bands typical of the *QC* molecule, with their intensity increasing with the *QC* concentration in the aerosol feed.

**Figure 3:**
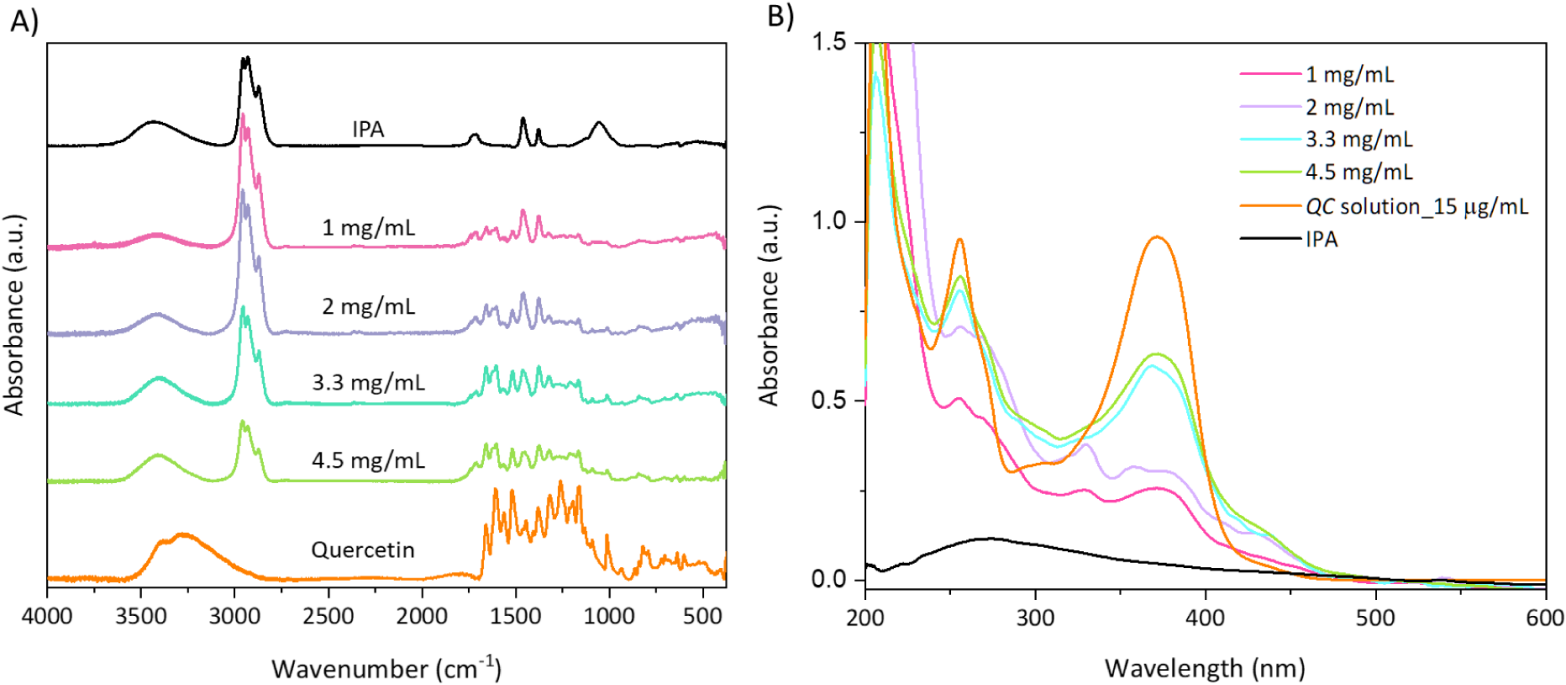
A) FTIR spectra of *QC*_NC_ coatings deposited at different aerosol quercetin concentrations (as reported on each curve). Spectra of *QC* and *QC*-free films (labeled as IPA) are reported for comparison. B) UV–vis spectra of 2 mL of MeOH after soaking *QC*_NC_ coatings deposited at different *QC* aerosol concentrations and of a *QC*-free (IPA) film. The spectrum of a 15 μg/mL *QC* solution in MeOH is included for comparison.

The UV–vis spectra were acquired using the same method used for the bilayers and are shown in Figure 3B. In this case, the characteristic *QC* bands are more clearly visible in samples deposited at a *QC* concentration higher than 3 mg/mL. By contrast, in the spectra of coatings deposited from aerosol solutions of 1 and 2 mg/mL, the bands relative to *QC* appear less defined and intense.

As shown in Table 3, the deposition rate of the *QC*_NC_ coatings is between 35 and 57 nm/min and increases with the concentration of *QC* in the aerosol feed solution. A similar trend is observed for the wettability of the composite films: they exhibit hydrophobic character with WCA values greater than 100°. For comparison, the deposition rate and WCA values of the *QC*-free IPA coatings were found to be 23 ± 5 nm/min and 94 ± 1° (see Figure S2 for corresponding water drop images), respectively. The increased hydrophobic character could reduce the interaction between the embedded *QC* and the food to be protected. On the other hand, the stability of quercetin within the coating could be enhanced, thereby prolonging the antioxidant effect. These considerations should be taken into account in the application of this approach to food packaging.

**Table 3:** Deposition Rate, WCA, Amount of *QC* Recovered from the Coatings (on 1.5 × 1.5 cm^2^ Silicon Substrates), and Experimental and Estimated Antioxidant Activity for the *QC*-Free Film and *QC*_NC_ Deposited at Different Aerosol Quercetin Concentration^a^

Sample	Deposition rate (nm/min)	WCA (°)	*QC* ^b^ amount recovered (μg/cm^2^)	Experimental^b^ antioxidant activity (%)	Estimated^b^ antioxidant activity (%)
IPA coating (*QC*-free)	23 ± 5	92 ± 4	ND	ND	ND
1 mg/mL	35 ± 7	103 ± 1	0.7 ± 0.2	4.7 ± 0.3	0.0 ± 0.9
2 mg/mL	32 ± 3	120 ± 3	1.2 ± 0.4	12.0 ± 0.8	0.3 ± 0.7
3.3 mg/mL	46 ± 6	124 ± 9	3.6 ± 0.8	12.7 ± 0.8	7.6 ± 0.5
4.5 mg/mL	57 ± 7	134 ± 8	3.1 ± 0.5	17 ± 1	5.6 ± 0.4

a The estimated antioxidant activity is that obtained from the calibration curve in Figure S3, for an amount of quercetin equal to the recovered amount.

b Values obtained onto 500 nm thick films.

The higher deposition rate obtained for the nanocomposite coatings accounts for the inclusion of *QC*, which adds building blocks to the growing film. To get further insights, SEM analysis was carried out, and the results are illustrated in Figure 4. The film deposited from IPA appears to be homogeneous, while the composite ones exhibit submicrometer-sized globular aggregates, which form during the deposition process and increase in size and number as the concentration of *QC* in the aerosol increases. The formation of such nanostructures is common when depositing films by aerosol-assisted plasma, and it is typically due to the solvent evaporation from the aerosol droplets, containing a solute that is solid at room temperature.^32,47^ In addition, as shown in Figure 4, the presence of *QC* in the film results in increased roughness compared to those obtained from IPA. The increased roughness accounts for the WCA values higher than 100°, even though in general there is not a direct correlation between roughness and WCA.

**Figure 4:**
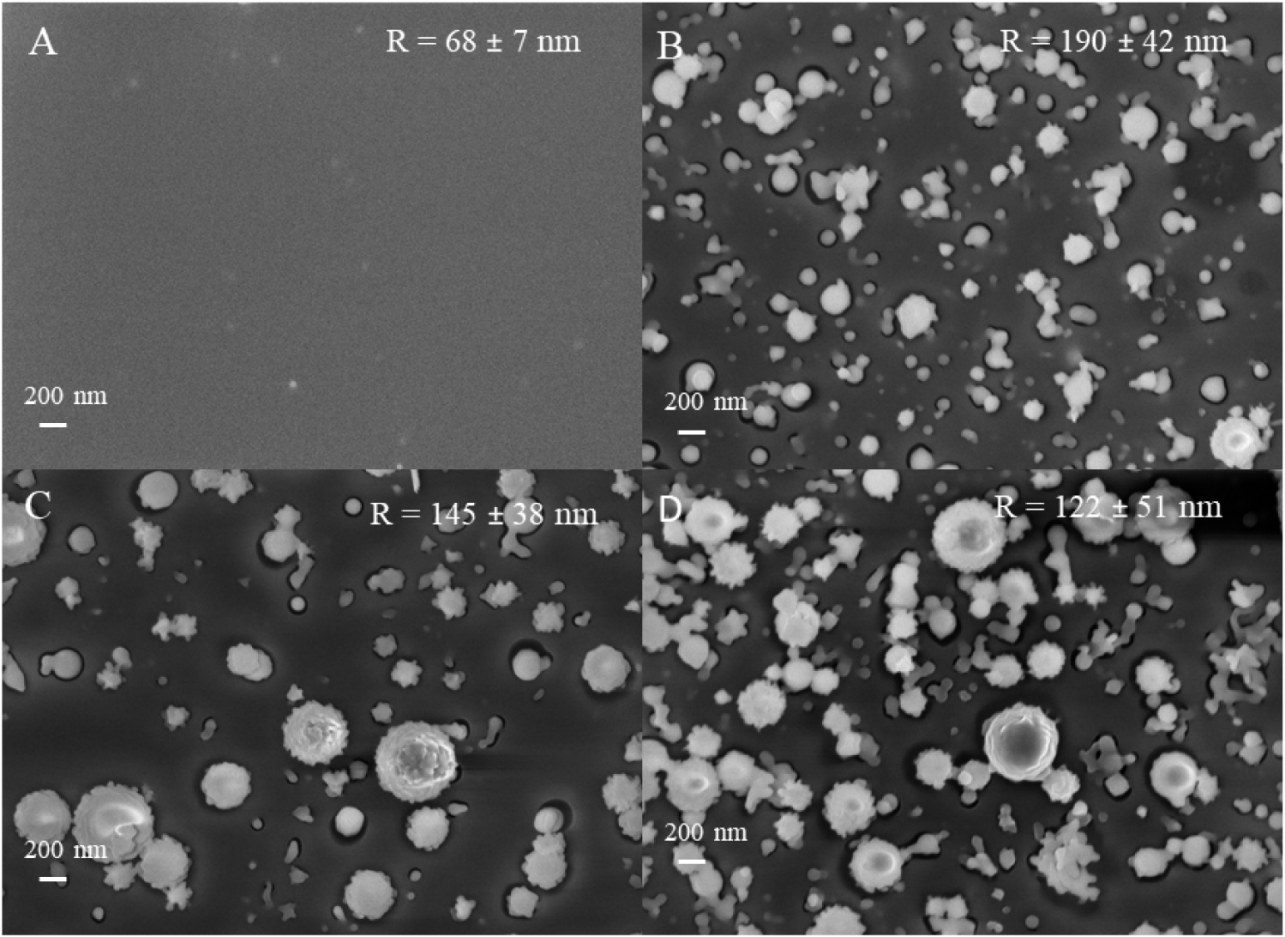
SEM images of (A) an IPA-based film and composite film plasma-deposited from aerosol *QC* solutions of (B) 1 mg/mL, (C) 3.3 mg/mL, and (D) 4.5 mg/mL *QC* in IPA. Roughness values, R, are reported for each sample.

The amount of *QC* present in 500 nm-thick films is given in Table 3. As shown in the table, the recovered amount ranges from 0.7 to 3.6 μg/cm^2^ and increases progressively with the *QC* concentration in the aerosol. However, for the 4.5 mg/mL sample, the recovered *QC* shows a slight decrease. This reduction may be attributed to the higher concentration of the starting solution, which can be more difficult for efficient nebulization. On the other hand, it is likely that, at the higher *QC* aerosol concentration, plasma can more easily promote the formation of quercetin oligomers, leading to a lower amount of *QC* recovered, but a significant increase in antioxidant activity. However, further molecular in situ diagnostics would be beneficial to demonstrate such hypothesis.

The antioxidant activity of the composite films was evaluated by means of the DPPH test; the data are presented in Table 3. As reported in Table 3, the antioxidant activity ranges between 4 and 17%, increasing with the concentration of *QC* in the solution, and, as for the bilayers, the measured antioxidant activity is higher than the estimated one.

## 5 Discussion

The results presented so far confirm that the plasma process can be a useful tool for embedding *QC* in a coating of potential use in active packaging, although its efficacy is limited in the case of the direct deposition process due to the low embedding efficiency of *QC* in *QC*_NC_. From the HPLC data, it appears clear that *QC* undergoes partial degradation during the plasma process, even though the measured antioxidant activity is higher than expected on the basis of the recovered *QC* amount. This effect is more evident in the double-step approach, where the initial amount of the cast *QC* is known. Some studies on the effects of cold plasmas on polyphenols have been reported in the literature, particularly in the context of food processing.^48–50^ In general, the reactivity of polyphenols toward plasma species varies; however, flavonoids tend to degrade more rapidly due to their strong ability to scavenge plasma-generated free radicals.^49,50^ The work of Kim et al.^48^ reports the effect of a DBD plasma on *QC* in methanol solutions, and despite the observed degradation, an enhancement of antioxidant activity has been found. In particular, the introduction of additional hydroxyl groups and carbonyl functionalities seems to increase the radical scavenging capability of the resulting intermediates. However, prolonged plasma treatment ultimately leads to a reduction in antioxidant potential due to the complete breakdown of the flavonoid structure. Consequently, the set of degradation intermediates generated by plasma treatment may exhibit an antioxidant activity higher than that of *QC* itself. This behavior may explain the results reported in Table 3.

To better understand the role of plasma in the present work, drop-casted *QC* was treated with DBD fed with different feeds, keeping constant V_pp_, frequency, and duration. The samples were then extracted with methanol by using the same procedure described in the previous sections. The UV–vis absorption spectra of the resulting solutions are reported in Figure 5.

**Figure 5:**
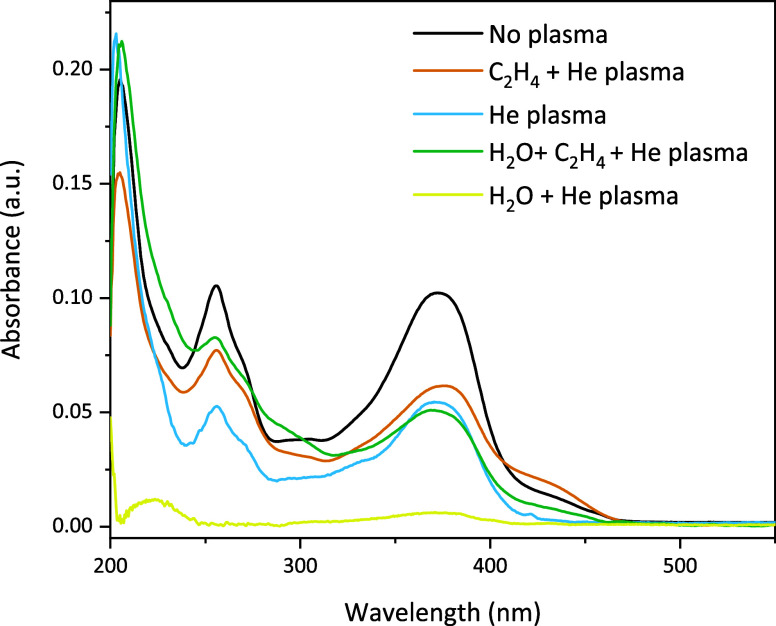
UV–vis absorbance spectra of solutions of 20 μg drop-casted *QC* in IPA samples dissolved in MeOH before and after exposure to different DBD for 10 min.

As observed, the absorbance of the characteristic peaks at 255 and 375 nm decreases for all plasma feeds investigated, indicating an alteration of the *QC* structure. The strongest effect is observed for the plasma feed containing only helium and water. When ethylene is added or when water is absent from the plasma, the effect becomes much less pronounced. This behavior can be attributed either to the absence of oxidative species generated from water (in the case of water-free feeds) or to a shielding effect caused by film deposition during the plasma process (in C_2_H_4_-containing feeds). This aspect is particularly noteworthy, as studies on plasma treatment of polyphenols have so far focused exclusively on nondepositing plasmas. Therefore, the interaction between antioxidants and deposited plasma discharges remains poorly understood and warrants further investigation. The present study represents the first contribution toward addressing this gap.

The same arguments can apply to the direct deposition of *QC*-containing coatings, as can be argued from the HPLC data. However, in this case, the feed does not contain water but IPA, the precursor of the embedding matrix. It can be supposed that during the deposition, the growing matrix at least partially protects quercetin. Further considerations were drawn. In comparison with previous studies,^33,34^ where it was demonstrated that the molecular structure of the chosen active molecule was mostly retained, in the present study, this does not apply for different reasons:

IPA is more volatile than water; hence, it does not shield *QC* from the plasma.

The solvent itself induces degradation of the *QC.*

*QC* is intrinsically less stable.

Concerning the possible toxicity of the formed byproducts, the literature gives some interesting hints. It has been shown that intentionally polymerized *QC* are biocompatible and promising for drug delivery.^51,52^ Plasma-induced transformations mainly yield compounds such as alphitonin and protocatechuic acid derivatives, which are considered safe and may provide health benefits.^53,54^ Additionally, process conditions may lead to the formation of methylated *QC* derivatives (e.g., isorhamnetin, rhamnetin, tamarixetin, azaleatin), which are naturally occurring compounds. Overall, while the literature suggests that *QC* by-products are likely safe, further studies are needed to fully assess the composition and safety of coatings.^48,49,55,56^

A further point that deserves discussion is the feasibility of such coatings on polymeric substrates used in food packaging. Thus, some preliminary tests have been carried out on PET for the promising *QC*_bilayer_. For comparison, a *QC*_casted_ film and a barrier layer film were deposited on the same substrate, a photograph of the materials was taken, and the WCA was evaluated. As shown in Figure S4, the *QC*_bilayer_ film was successfully deposited onto PET without damaging the substrate. The coatings appear slightly yellowish, and the total transmittance in the visible spectral region is reduced. Figure S5 shows no major changes in the WCA of bare and coated PET. The change in color and transparency would suggest that a more suitable substrate in packaging would not be the transparent protecting film but more likely the pad in contact with the food. Though the coatings and processing conditions should be better optimized, the preliminary results are encouraging for the application of plasma processes to the deposition of antioxidant coatings for food packaging.

An important issue in food packaging is gas permeability. According to existing literature, the addition of polyphenols, such as *QC*, can influence the permeability of water vapor and oxygen, but these variations are usually limited to the same order of magnitude and are highly dependent on the polymer matrix.^17,57,58^ In this study, the developed coatings are hundreds of nanometers thick. At this scale, the intrinsic barrier contribution of this type of coating is less significant than that of the underlying substrate. However, the application of these *QC*-functionalized coatings onto high-barrier substrates could lead to advanced multilayer systems, where antioxidant activity and gas-barrier properties are combined synergistically to maximize food shelf life.

## 6 Conclusion

Two different strategies were employed to prepare quercetin-containing plasma-deposited composite coatings for potential application in active food packaging: (i) a bilayer approach, in which a quercetin layer was drop-cast onto the substrate and subsequently covered with a plasma-deposited coating; and (ii) the direct deposition of a quercetin-containing nanocomposite coating—from an aerosol of its solution in IPA.

Material characterization confirmed the successful incorporation of quercetin in the coatings using both strategies, although the direct deposition method limited the amount of embedded quercetin to only a few micrograms. Furthermore, comparison between HPLC and UV–vis absorption spectroscopy analyses of recovered quercetin indicated partial degradation of the molecule, partly attributable to its known interaction with the alcohol used as feed in this work. However, an important role of plasma in inducing alteration of quercetin has been demonstrated.

This study highlights for the first time the different effects of nondepositing and depositing plasmas on quercetin processing.

Despite the observed alteration of the active compound, significant antioxidant activity was retained, demonstrating the potential suitability of these coatings for food packaging applications. Future work will focus on optimizing plasma parameters to minimize *QC* degradation and exploring the scalability of the process for industrial applications.

## Supplementary Material



## Notes

Table of barrier coating thickness as a function of deposition time (Table S1); HPLC UV-vis chromatogram of quercetin detected at 370 nm (Figure S1); WCA images of A) barrier coating, B) IPA coating (*QC*-free) and *QC*_NC_ coatings, C) 1mg/mL, D) 2 mg/mL, E) 3.3 mg/mL, and F) 4.5 mg/mL (Figure S2); calibration curve of antioxidant activity of quercetin tested with DPPH in MeOH (Figure S3); A) picture and B) visible transmittance for the PET substrate and barrier layer, *QC*_casted_ and 25 μg_600 nm bilayer deposited on PET (Figure S4); WCA images of the A) PET substrate; B) barrier layer; C) *QC*_casted_, and D) 25 μg_600 nm bilayer deposited on PET (Figure S5) (PDF)
