# Supplementary material for: Aerosol-Assisted Plasma Deposition of Quercetin-Containing Coatings for Active Packaging Applications
Source: ACS Omega. 2026 Jul 8;11(28):42412–21. doi: 10.1021/acsomega.6c03125 (PMC13393205; doi:10.1021/acsomega.6c03125)
Supplement: Supplementary file 1 [file ao6c03125_si_001.pdf]

Supporting Information

Aerosol assisted plasma deposition of Quercetin containing coatings for active packaging

Angelica Maria Lanza[1,2], Antonella Milella[1,2], Nicoletta De Vietro[3], Giovanna Mancini[3], Fabio Palumbo[2], Donatella Nardiello[4], Francesco Fracassi[1,2], Carlo Zambonin[3], Pietro Favia[1,2]*

1 Department of Chemistry, University of Bari "Aldo Moro", via Orabona 4, 70125 Bari.
2 Institute of Nanotechnology, Bari Site, CNR, c/o Department of Chemistry, University of Bari "Aldo Moro", via Orabona 4, 70125 Bari.
3 Department of Biosciences, Biotechnology and Environment, University of Bari "Aldo Moro", via Orabona 4, 70125 Bari.
4 Department of Agriculture, Food, Natural resources and Engineering (DAFNE), University of Foggia, via Napoli 25, 71122 Foggia, Italy
*corresponding Author: fabio.palumbo@cnr.it

Tab. S1. Table of barrier coating thickness as a function of deposition time.

**Deposition time (min)**	**2**	**4**	**6**	**10**
**Thickness (nm)**	193 ± 14	371 ± 40	661 ±100	994 ± 80

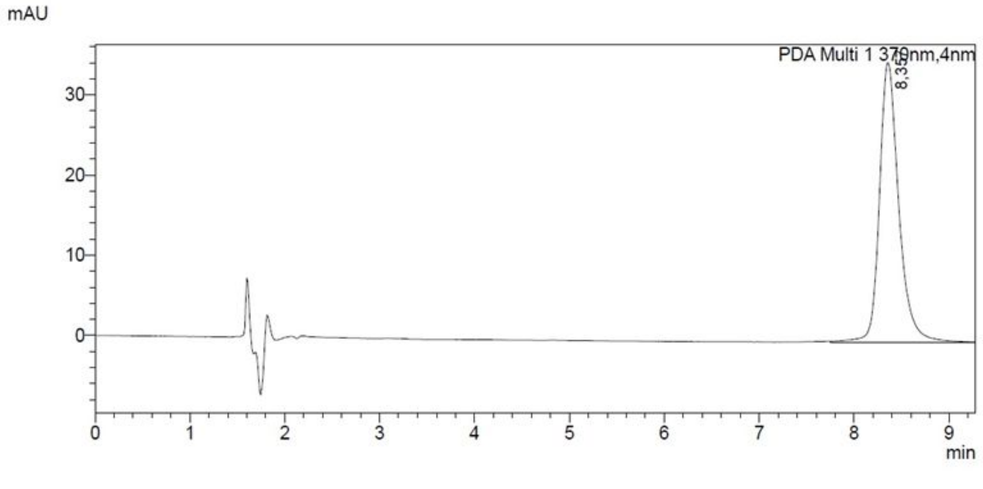

Fig. S1. HPLC UV-vis chromatogram of quercetin detected at 370 nm

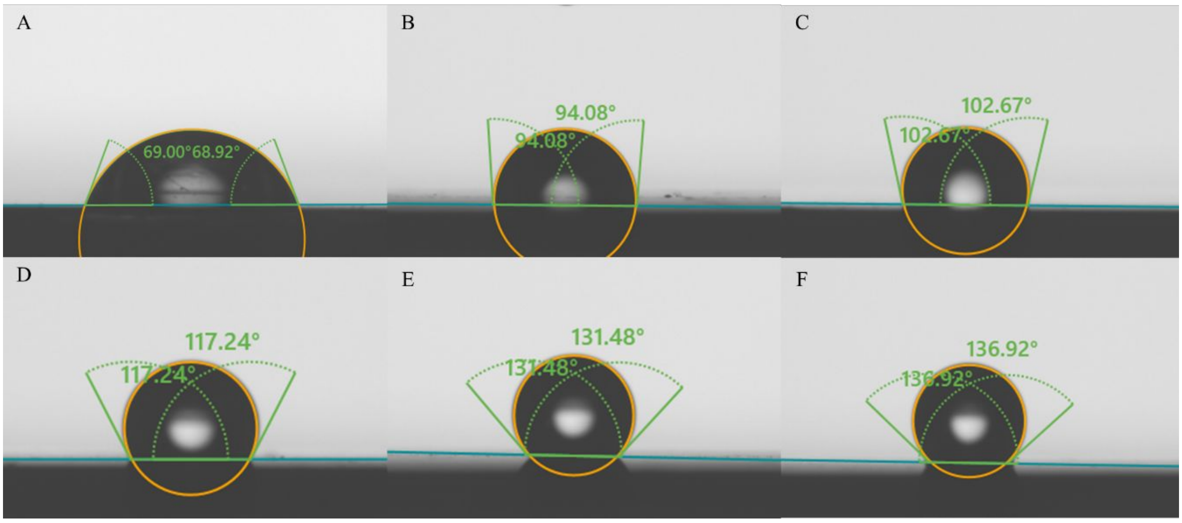

Fig. S2. WCA images of A) Barrier coating; B) IPA coating (*QC*-free) and QC_{NC} coatings: C) 1mg/mL; D) 2 mg/mL; E) 3.3 mg/mL and F) 4.5 mg/mL.

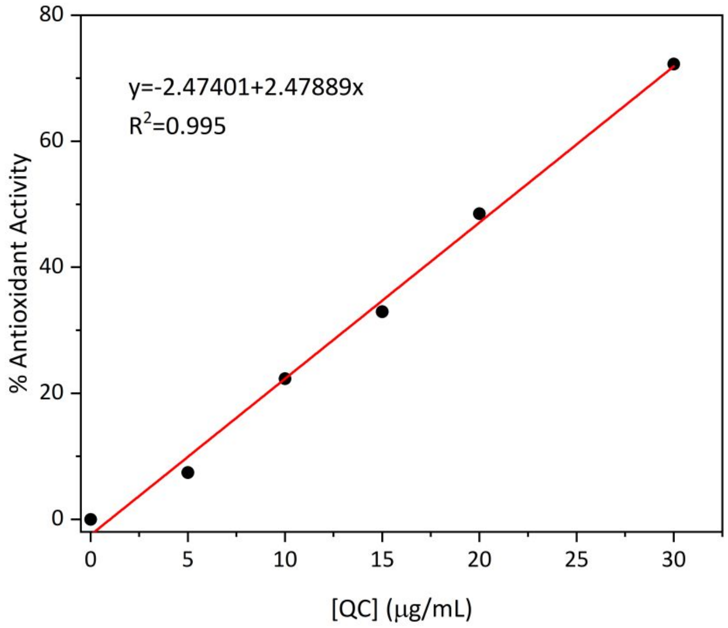

Fig. S3. Calibration curve of antioxidant activity of quercetin tested with DPPH in MeOH.

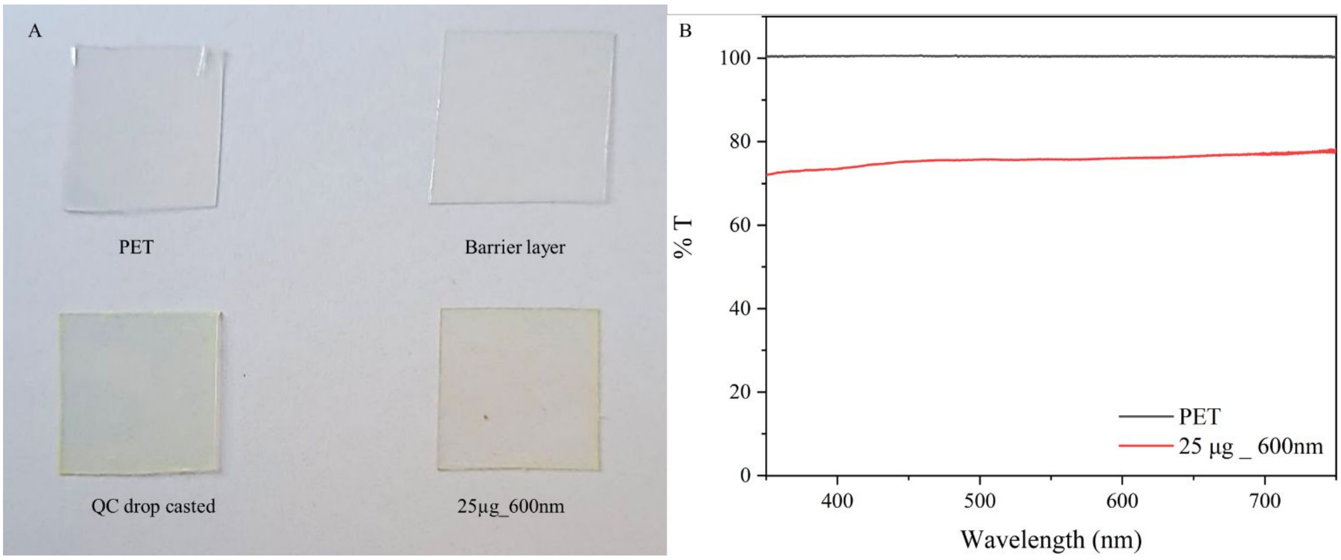

Fig. S4. A) photo of PET substrate and, barrier layer, QCcasted and 25µg_600nm bilayer deposited on PET. B) graph showing the percentage of transmittance for PET substrate with and without bilayer

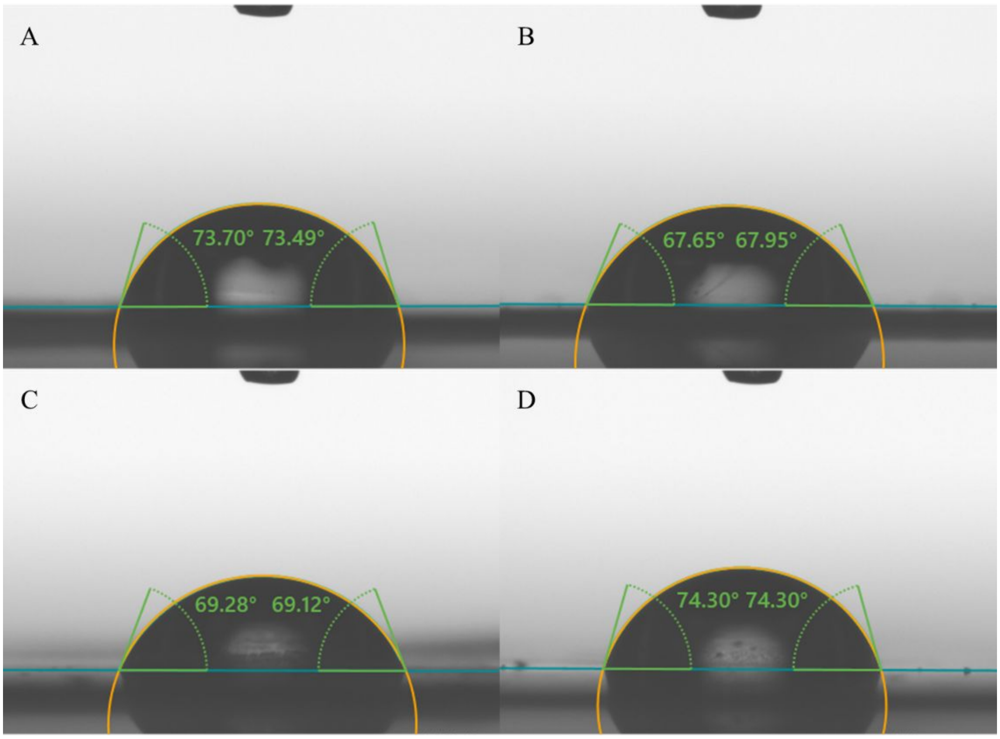

Fig. S5. WCA images of A) PET substrate; B) barrier layer; C) QC_{casted} and D) 25µg_600nm bilayer deposited on PET.
